# King's College Hospital

**Published:** 1909-07-24

**Authors:** 


### HOSPITAL ADMINISTRATION.

### CONSTRUCTION AND ECONOMICS.

### KING'S COLLEGE HOSPITAL.

THE KING AND QUEEN AT DENMARK HILL.

Tuesday was one of the most beautiful days of the year. The weather was delightful, and the authorities and people of Camberwell deserve great credit for the beauty and taste displayed by the decorations which fitly framed the genuine enthusiasm with which they welcomed their Majesties as they passed through the district. It was a bold policy to determine to move King's College Hospital from Lincoln's Inn Fields to Denmark Hill, but the wisdom of the step was undoubted, and the proof that public opinion has been behind the authorities is magnificently expressed by the receipt already of £230,000 towards the £300,000 required. The fact that so large a sum had been raised encouraged the thoughtful to believe that those at present responsible for the administration of the affairs of King's College Hospital were worthy of the fullest trust, and that they possessed the gift of administration and capacity to control and successfully carry through the great enterprise involved by the policy of removal. This confidence was abundantly justified and increased, if possible, by the excellence of the arrangements for the ceremony on Tuesday.

It would be interesting to know what is the total number of foundation stones which His Majesty the King has officially laid during his life. Our experience of these ceremonies in the past has left an impression in our minds that, however important the commencement of a new building may be, the ceremony of laying the foundation stone is one which wise people may contentedly avoid. Tuesday's ceremony is the exception. The Hon. W. F. D. Smith, M.P., Mr. Charles Awdry, and those associated with them, who were responsible for the arrangements, deserve most hearty congratulations and cordial acknowledgments. The organisation under their direction was so perfect that everybody present must have been pleased and glad that they came.

The pavilion was an enormous one, kept full of fresh air, and the seating had been so arranged that everybody had an excellent view and ample space, so that there was no crowding, or discomfort, or disappointment. Everyone was able to find their seat, and the whole of the vast audience showed commendable punctuality in taking their places, so that everything was in perfect order long before the hour fixed for the arrival of their Majesties. The Guards' Band, which was excellent, gave pleasure to all present, and the grouping of the nurses, with Miss Ray, the sister-matron, in the centre, was not the least attractive feature of an assemblage which was brightened by the gay colours of the hoods and gowns of the graduates, and by the beauty of many of the dresses, displaying a great variety of colours, worn by the ladies present. One feature of the initial proceedings before the ceremony was the amusing way in which a small boy patient dressed from head to foot in red, when brought on to the platform by one of the Sisters, who explained to him how he was to present a bouquet to Queen Alexandra, gradually shed his shyness and proceeded to adopt all sorts of unconscious attitudes to the great amusement of those present. The attendance included the professors and teachers, the physicians and surgeons, and several eminent authorities now living who have done so much to add lustre to the reputation of King's College and its Hospital; the Mayors of Camberwell, Southwark, and Deptford, the Masters of the Drapers' and Clothworkers' Companies, the Chairmen of St Mary's and the Middlesex Hospitals, the President of the Royal College of Surgeons, the Portuguese Minister, Lord Northcote, Sir Savile Crossley, Sir W. H. Watson Cheyne, Sir Henry Burdett, the Master of the Temple, the Secretary (Captain Tunnard), and the Secretary of the Removal Fund (Mr. George Heyer).

We have said that the organisation of the preliminaries was perfect, and the same adjective may properly be applied to the ceremony itself. Every word, as it fell from the lips of each speaker, was distinctly audible to all present. This in itself is a pleasant novelty which deserves recognition. The Bishop of Southwark prepared the audience by explaining to them the part they had to take in the ceremony, and their responses were hearty and added something to the completeness of the proceedings. Mr. Smith read the address exceedingly well, the Bishop's service possessed the merit of brevity and point, and His Majesty the King made, as those who read it will admit, a most excellent speech. The real interest of the ceremony, however, centred in the foundation stone laying itself. In performing this act His Majesty made it the occasion to show all present that if a thing is worth doing it is worth doing well. It was remarkable to watch the continuous interest of the King and his insistence upon the most accurate fulfilment of every item of the ceremony with perfect orderliness and in the most perfect manner. He laid the mortar with infinite care; he watched and directed the laying of the stone itself; he measured and approved it, and tapped it with the mallet with a dignity and gravity which must have impressed everybody. Then turning to the audience, with bowed head he uttered the words: "In the name of the Father, and of the Son, and of the Holy Ghost, I declare this stone to be well and truly laid."

King Edward is, in a very real sense, the Father of his people, and the confidence which every class of his subjects, from the highest to the lowest, place in their Sovereign is well deserved. In all things and on all occasions he is kingly, and there is a thoroughness about His Majesty's discharge of his public duties which gives them not only a special charm but a special value, in the sense that on each occasion his actions drive home to all who witness them the soundness of the principle that everything that is worth doing is worth doing well.

King's College Hospital, and all who are responsible for its administration, are to be congratulated upon the success of Tuesday's proceedings. They may be taken as a model to be followed by all who have in future to organise similar ceremonies, for it is but just to say that they were in all respects perfect. We hope that the remaining £70,000 required to complete the building fund will speedily be forthcoming, for gentlemen who have shown the administrative gifts which the present position of the building fund and the organisation just described prove, deserve the confidence and support of all who have it in their power to give liberally to the support of hospitals, especially when they are careful before parting with their money to obtain guarantees that those who will have the expenditure of such gifts may be trusted to administer them with the maximum of economy and efficiency.

### The Address to the King.

The following is the Address delivered by the Hon. W. F. D. Smith, Chairman of the Committee, to Their Majesties the King and Queen:—

"May it please Your Majesties,—I am privileged on behalf of the Removal Committee of King's College Hospital to offer our humble duty to Your Majesties, and to express our deep gratitude for the great honour which Your Majesties have conferred upon the Hospital by your presence upon this occasion.

"Six years ago the Committee of King's College Hospital were called upon to decide whether they would spend a large sum of money upon the old Hospital in order to bring it up to modern requirements, or whether they would undertake the construction of a new Hospital in South London, where a poor and populous district was sadly in need of Hospital accommodation.

"The Committee of 'King Edward's Hospital Fund' were strongly in favour of a removal scheme, and after a full enquiry into the changed conditions of the population surrounding the old Hospital, caused by the clearance of crowded and insanitary areas, and on account of the fact that numerous Hospitals already existed in a comparatively small area north of the river, it was decided to adopt the policy of the Fund and to construct a new Hospital in South London. This building when completed will give accommodation for 600 beds, and ample provision for all the special departments now necessary for the efficient working of every general Hospital.

"The site extends to over twelve acres, and in addition land on the south side, to the extent of more than thirty acres, has been acquired, partly by private liberality and partly by public funds, for a people's park, so that the Hospital will for ever have the advantage of an adjoining open space larger than any enjoyed by similar institutions in London.

"The Committee of 'King Edward's Hospital Fund have given munificent donations, amounting in all to £27,000, to the Removal Fund, which has now reached a total of over £330,000. It is probable that the original estimate of the cost of removal will be somewhat exceeded, but the Committee have every hope that private generosity will provide them with sufficient funds to complete the building now undertaken, without trenching upon the capital sum realisable by the sale of the old Hospital site and building, but which it is of vital importance to retain for future endowment.

"As Your Majesties will have observed, the building of the out-patient, casualty, and some of the special departments is already far advanced, and it is hoped that in twelve months the whole of the building now contemplated will have been begun.

"I now have the honour to request Your Majesty to lay the foundation stone of the new King's College Hospital, and in so doing to perpetuate the connection with Your Majesty's Throne and Family which our Hospital by its style and title has the honour of commemorating."

### The King's Speech in Reply.

His Majesty made the following reply:—

"I thank you most heartily, on behalf of the Queen and myself, for your loyal address and for the cordial welcome you have offered to us. It gives me great pleasure to come to lay the foundation-stone of the new building of the hospital with which the Sovereign's name is connected, and in whose past work I have taken so deep an interest. For many years King's College Hospital has ministered to the necessities and alleviated the sufferings of thousands of my subjects in the centre of this crowded Metropolis. It was specially and closely associated with the great work of Lord Lister, who, in introducing the antiseptic methods in surgery, rendered an inestimable service to England and to all mankind. I remember also that your hospital sent out some of the most distinguished of its staff to do generous work among the sick and wounded in the South African war. Your committee's decision to abandon the old building and to remove the scene of your labours to a poorer and more populous district, where the need for a hospital was great and the field for its work wide, was a bold step, but I believe it was a wise and right one, and I congratulate the medical and nursing staff on the great opportunity thus given them for increased usefulness. The generous response made to the committee's appeal for funds shows how their decision has been appreciated by the public. I am glad that the fund associated with my name favoured the scheme in its inception and has given material assistance in carrying it out. I am confident that other generous donors will come forward to supply the money needed to complete a spacious and well-equipped building. I can conceive no worthier object for benevolence than this. I assure you of my continued interest in your hospital and of the sympathy with which I shall watch its future progress. I cannot doubt that the blessing of Almighty God will attend the work to be done by it in its new home."

After the Bishop of Southwark had offered up prayer, His Majesty laid the foundation-stone; the Benediction was pronounced by the Bishop, and afterwards a number of presentations were made to His Majesty.

